# Parametric study of a bubble removing device for hemodialysis

**DOI:** 10.1186/s42490-023-00069-3

**Published:** 2023-04-01

**Authors:** Poonnapa Chaichudchaval, Nunthapat Fuangkamonvet, Supajitra Piboonlapudom, Teeranoot Chanthasopeephan

**Affiliations:** Department of Mechanical Engineering, Faculty of Engineering, King Mongkut’s University of Technology Thonburi, Bangkok, 10150 Thailand

**Keywords:** Hemodialysis, Bubble, Swirl Number, Taylor Number

## Abstract

**Background:**

This paper sets out to design a device for removing bubbles during the process of hemodialysis. The concept is to guide the bubbles while traveling through the device and eventually the bubbles can be collected. The design focuses on the analysis of various parameters i.e. inlet diameter, inlet velocity and size of the pitch. The initial diameters of Models 1 and 2 have thread regions of 6 and 10 mm, respectively. Parameters: Swirl number, Taylor number, Lift coefficient along with pressure field are also implemented.

**Results:**

Based on computational fluid dynamics analysis, the bubbles' average maximum equilibrium position for Model 1 reached 1.995 mm, being greater than that of Model 2, which attained 1.833 mm. Then, 16,000 bubbles were released into Model 1 to validate the performance of the model. This number of bubbles is typically found in the dialysis. Thus, it was found that 81.53% of bubbles passed through the radial region of 2.20 ± 0.30 mm. The appropriate collecting plane was at 100 mm, as measured from the inlet position along the axial axis. The Taylor number, Lift coefficient, and Swirl number proved to be significant parameters for describing the movement of the bubbles. Results were based on multiple inlet velocities. It is seen that Model 3, the improved model with unequal pitch, reached a maximum equilibrium position of 2.24 mm.

**Conclusion:**

Overall, results demonstrated that Model 1 was the best design compared to Models 2 and 3. Model 1 was found capable of guiding the bubbles to the edge location and did not generate extra bubbles. Thus, the parametric study, herein, can be used as a prototype for removing bubbles during the process of hemodialysis.

## Background

In general, bubbles in hemodialysis are defined as bubbles of diameter less than 200 µm [1]. Consequently, these bubbles can cause pulmonary embolism, and was first discovered during bypass surgery [2, 3]. When the capillaries of the lungs are full of bubbles, they can interrupt the circulation of the blood, and can cause damage to the patients.

During the process of hemodialysis, blood is removed from the body and cleaned in a dialyzer, before being returned; during this process, bubbles are found [4, 5]. Contamination of air in the blood is also a cause of hemodialysis problems [6–8]. The dialyzer, part of the hemodialysis machine for transferring waste, and purifying the blood is the main section that involuntarily generates bubbles [1]. It is noted that the surrounding fiber tubes of the dialyzer provide a greater volume of air than the center tubes. As a result, the process of elimination is found to be inefficient. Because of the lower buoyancy force of the bubbles, the fundamental bubble trap of the dialysis machine is also inoperative, depending on the size of the bubbles. The bubbles, which cannot move upward to the blood surface, have high surface tension, and can cause bubble foam [1, 9]. Hence, ultrasound is used to detect when bubbles appear in the system, though miniature bubbles cannot be detected [10, 11]. Therefore, one of the problems patients encounter undergoing dialysis is the formation of bubbles. Removing bubbles from the circuit is essential.

To improve the effectiveness of the process of hemodialysis, the study of bubbles, including arterial pressure and blood velocity, has been undertaken [9]. Numerical analysis has been conducted to analyze the blood flow in arteries [12]. Thus, computational fluid dynamics (CFD) was carried out to inspect the characteristics of bubbles; the purpose being to guide the direction of the bubbles along the thread region so that the bubbles can be collected in a specific region. It is acknowledged that the bubble trap operates for both 2-dimensional and 3-dimensional simulations [13, 14].

Gas liquid separation has been explored in various fields, including the nuclear industry [15, 16]. To separate air from water, an air core is formed. The efficiency of separation is based on the outlet angle of swirl vanes, volumetric flow rate and the density of the working fluid. To accomplish our work, to get rid of the bubbles, six forces have been undertaken: buoyancy force (F_B_), drag force (F_D_), Saffman lift force (F_L_), added mass force (F_A_), Basset history force (F_BH_), and pressure gradient force (F_P_) [17]. According to Newton’s law, such forces generate the overall motion of the bubbles, as expressed in Eq. (1):

1 $${\text{m}}_{\text{p}}\frac{\text{du}}{{\text{dt}}}= {\text{ F}}_{\text{B}}{\text{+F}}_{\text{D}}{\text{+F}}_{\text{L}}{\text{+F}}_{\text{A}}{\text{+F}}_{\text{BH}}{\text{+F}}_{\text{P}},$$

The four major forces i.e. F_B_, F_D_, F_L_, and F_A_ are characterized by circular motion. Consequently, the balance of forces in the radial axis for the bubbles flowing in the blood is defined as in Eqs. (2) and (3):

2 $${\text{F}}_{\text{B}}\left(\text{sin}\theta\right)\left({\text{ + F}}_{\text{L}}-{\text{F}}_{\text{A}}\right)\text{ = 0}$$

3 $$\begin{aligned}{\mathrm F}_{\mathrm B}={\uprho}_1{\text{V}}_{\text{b}}\text{g sin}\theta ; & {\mathrm F}_{\mathrm L}={\uprho}_1{\text{V}}_{\text{b}}\upomega^2{\text{r}}_{\text{e}}\left(2{\text{C}}_{\text{L}}\right)\text{; }&{\mathrm F}_{\mathrm A}={}{\uprho}_1{\text{V}}_{\text{b}}\upomega^2{\text{r}}_{\text{e}}\left({\mathrm C}_{\mathrm A}+\text{1}\right)\end{aligned}$$

Where ${\uprho}_1$ is the blood density, ${\text{V}}_{\text{b}}$ is the volume of bubble, g is the gravitational acceleration, $\uptheta$ is the angle position of the bubble measured from the horizontal axis, ω is the angular velocity of the fluid, r_e_ is the equilibrium position, C_A_ is the added mass coefficient, and C_L_ is the coefficient of the lift force.

Thus, the radial distance of the bubbles from the center of the device was derived (Eq. 4). This distance is known as the equilibrium position (${\text{r}}_{\text{e}}$). The values of the equilibrium positions can change due to the position of the angle of the bubbles. Thus, the maximum values of the equilibrium positions, top and bottom positions, are taken into consideration. Equation (4) indicates the distance at that position [17]:

4 $${\text{r}}_{\text{e}}{ } = \frac{\text{-g}}{{\upomega }^{2}\left[{2}{\text{C}}_{\text{L}}-\left({\text{C}}_{\text{A}}+ \text{1} \right)\right]},$$

where r_e_ is the equilibrium position, C_A_ is the added mass coefficient, C_L_ is the lift force coefficient, and ω is the angular velocity of the fluid.

Parameters that involve the simulation of the bubbles include: Reynold’s number, Swirl number, Strouhal number, Taylor number, and Weber number. The Swirl number indicates the swirling flow that is the ratio of the angular momentum's flux to the axial momentum's flux. When the Swirl number is more than 1, such an outcome can cause the formation of bubbles, which is not desirable. The Swirl number is calculated for each cross-section plane via integration, as demonstrated in Eq. (5):

5 $$\mathrm{SW}=\frac{\int{\mathrm{rv}}_{\uptheta}\overset{\rightharpoonup}{\mathrm v}\cdot\mathrm d\overset{\rightharpoonup}{\mathrm A}}{\overline{\mathrm r}\int{\mathrm v}_{\mathrm z}\overset{\rightharpoonup}{\mathrm v}\cdot\mathrm d\overset{\rightharpoonup}{\mathrm A}},$$

where SW is the swirl number, r represents the radial distance, and $\overset{\mathrm{-}}{\text{r}}$ is the ratio of the cross-section area to the perimeter. $\overset{\rightharpoonup}{\mathrm v}$ is the unit vector for velocity, v_z_ is the axial velocity and ${\mathrm{v}}_{\uptheta }$ is the tangential velocity. $\overset{\rightharpoonup}{\mathrm A}$ means the cross-section area.

The Reynold’s number describes the characteristics of the blood flow passing through the bubbles. The Swirl number indicates the swirling flow, which is the ratio of the angular momentum's flux to the axial momentum's flux. A Swirl number, greater than 1, can cause the formation of bubbles. The Strouhal number presents the split in velocity called vortices. The appropriate Strouhal number is less than 1, for the common condition. This dimensionless number is the relationship between the radius of the bubble (R_b_) and the maximum equilibrium position (r_e_) [17].

The Taylor number describes the characteristics of the bubbles in the swirling flow. Thus, the Taylor number combines the phenomena of both Reynold's number and Strouhal number, so it represents the radial distance of the bubbles. Reynold’s number presents the flow properties and the Strouhal number presents the vortices. Accordingly, the Taylor number is derived, as in Eq. (6):

6 $$\text{Taylor } = \frac{{\left({2}{\text{R}}_{\text{b}}\right)}^{2}\upomega }{\upsilon },$$

where R_b_ is the radius of the bubbles, $\upomega$ is the angular velocity of fluid, and *υ* is the kinematic viscosity of the fluid.

The dissociation of deformation can be explained by the relationship between the Weber number and Ohnesorge number [18]. The lower values of both dimensionless variables result in the least deformation.

## Results

In Table 1, it was seen that the diameter of the bubbles could affect the position of maximum equilibrium. Moreover, the bubbles: 200 µm in diameter had moved farther from the center than the bubbles 10 µm in diameter. The increase in the thread length region had not affected the results of the simulation.

**Table 1:** Maximum equilibrium position (r_e_) of the different bubble diameters and thread length regions

**Model**		**r**_**e**_** (mm)**	
		**L**_**thread**_** = 90 mm**	**L**_**thread**_** = 180 mm**
Model 1	d_b_ = 10 µm	1.995	1.917
	d_b_ = 200 µm	2.269	2.333
Model 2	d_b_ = 10 µm	1.883	1.867
	d_b_ = 200 µm	2.223	2.218

### Effect of initial diameter

**Fig. 1:**
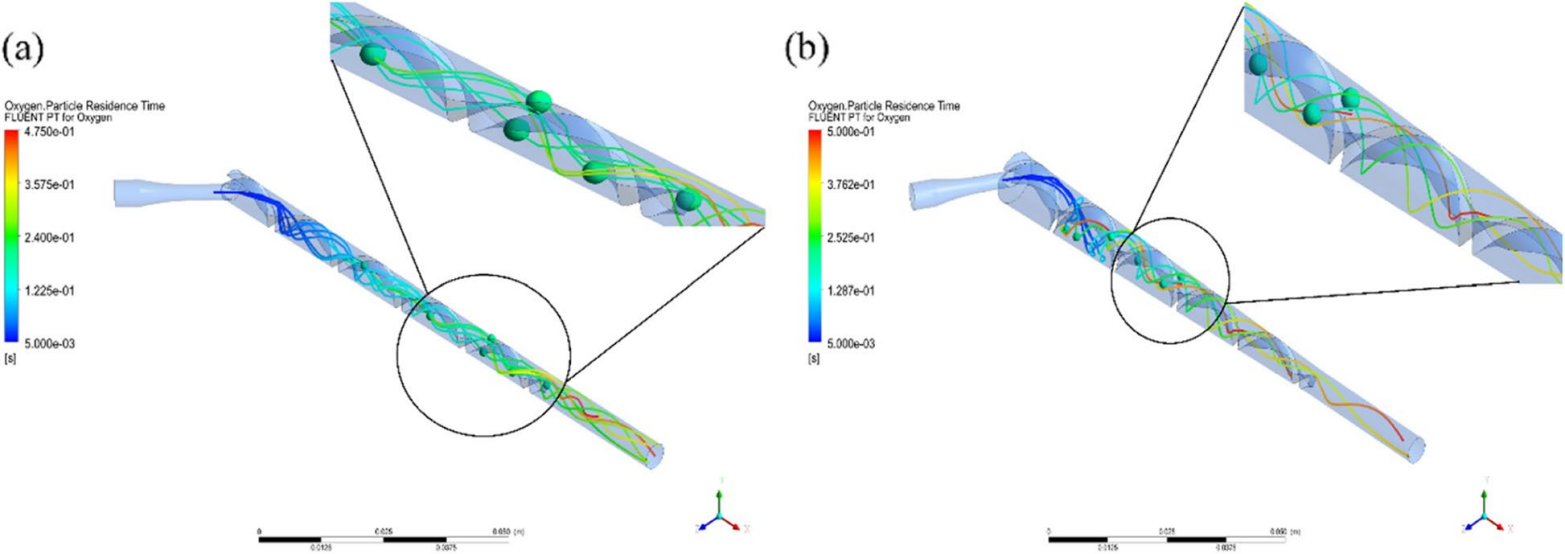
Simulation results of the even-pitch models

As Model 1 and Model 2 had different initial diameters, Fig. 1 showed the different simulation results when the bubbles traveled along the two models. The difference between Models 1 and 2 was the initial diameter. In other words, the front cross sections of the devices were different. Model 1 was 6 mm in diameter and Model 2 was 10 mm in diameter. This cross-section could change the thread angle of the design model even though the pitch length for both models was the same. Subsequently, the thread angle of Model 1, as measured from the axial axis of the device, was less than that of Model 2. When the direction of the thread angle and the inlet pipe coincided, the fluid flowed smoothly. Hence, the direction of the inlet pipe and thread angle of Model 1 was seen to promote the forward flow more than the circular flow.

**Fig. 2:**
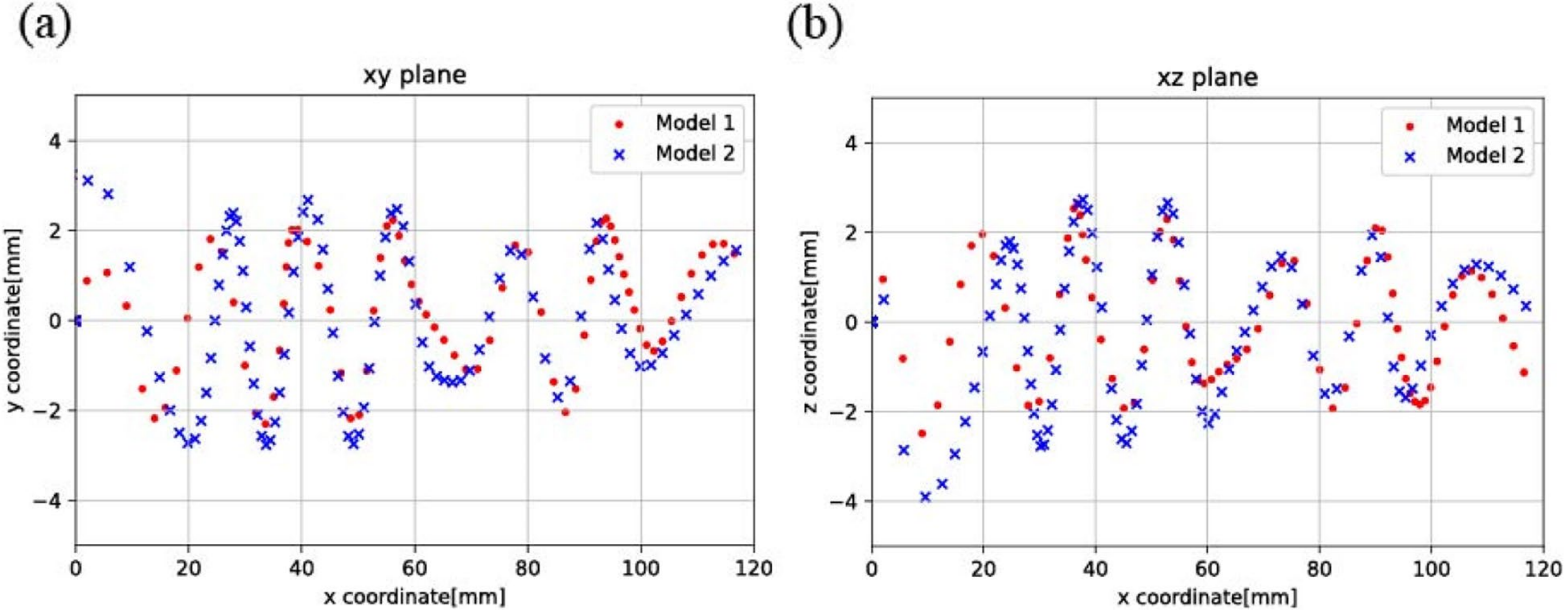
Position of the bubbles as observed in the different planes: (a) xy plane and (b) xz plane

In Fig. 2 (a, b), the discrete phase model (DPM) method was carried out. After the bubbles passed through the thread region, the bubbles transformed into a swirling flow pattern and continued swirling throughout the thread region and non-thread region. The swirling flow pattern remained in the area where no thread was presented.

Models 1 and 2 with their different inlet diameters, produced different angular velocities, though the characteristics of the bubble movements of both models were found to be similar. Subsequently, the maximum equilibrium position i.e. the position of the bubbles as measured from the center of the plane, for Model 1 was found to be 1.995 mm, which proved to be greater than that of Model 2 being 1.833 mm.

**Fig. 3:**
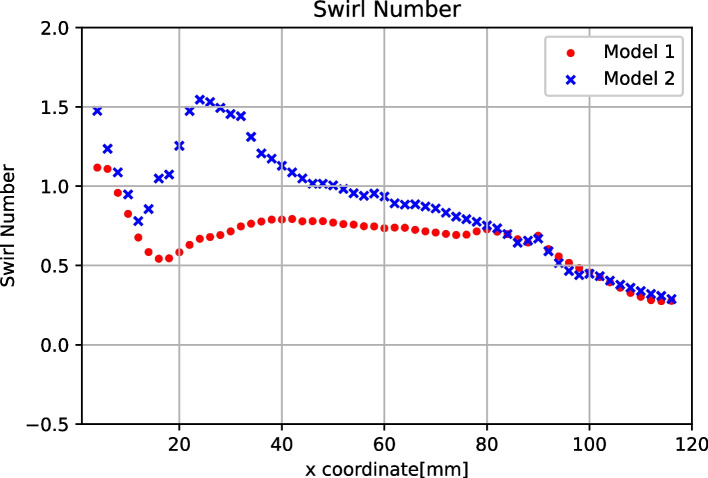
Swirl numbers along the axial axis of the equally pitched Models 1 and 2

As shown in Fig. 3, the Swirl number for Models 1 and 2 were determined. Based on Model 1, the average Swirl number proved to be 0.736 for the thread region and 0.425 for the non-thread region. Model 2, however, revealed a higher average Swirl number in the thread region i.e. 1.028 while the average Swirl number in the non-thread region was 0.426. Lift coefficients were also calculated for both Models 1 and 2, being 0.609 and 0.184, respectively.

### Bubble removal device

Of the two models, results of the simulation showed that the majority of the bubbles in Model 2 appeared closer to the center than those in Model 1. Therefore, the edge of the thread of the device was selected as the collective region for the bubble collector. Model 1 was then selected to perform an analysis of the bubbles. In a typical dialysis situation approximately 16,000 bubbles are presented [4]. Subsequently, Model 1 was similarly tested where 16,000 bubbles were released through the inlet of the device model; 8,000 bubbles were 10 µm in diameter, and 8,000 bubbles were 200 µm in diameter.

**Fig. 4:**
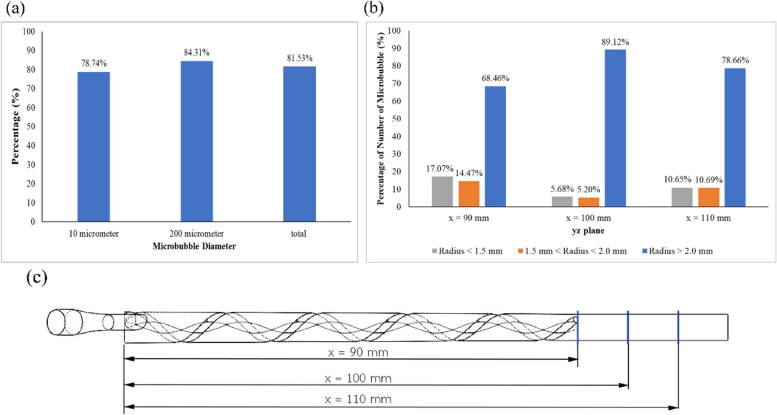
**a** Percentage of bubbles with different diameters passing through the radial distance of 2.204 ± 0.297 mm**, b** Percentage of bubbles located at different regions on the three planes, and (**c**) Three planes were selected to observe the number of bubbles

In Fig. 4 (a), 81.53% of the bubbles flowed through the region located at 2.204 ± 0.297 mm. The number of bubbles with a diameter of 10 µm that passed through this region: the target region proved to be less than the number of bubbles 200 µm in diameter. 78.74% of the bubbles 10 µm in diameter were mostly seen in the area where the average maximum position from the center was 2.12 ± 0.271 mm. In contrast, the average maximum equilibrium position of the bubbles 200 µm in diameter was found to be 2.29 ± 0.30 mm. The success rate of guiding the bubbles to the target region was 84.31%.

In Fig. 4(b, c), the x y z planes, and the number of bubble locations at each plane is presented. The yz planes, which are considered collecting planes, are located along the axial axis: where x = 90, 100, and 110 mm. The most efficient plane for the collection of bubbles was at 100 mm. The radial position of the bubbles was then analyzed via DPM; the greatest value proved to be 89.12%, which is close to the device’s wall, at the yz plane (100 mm).

### Effect of inlet velocity on the position of the bubbles

**Fig. 5:**
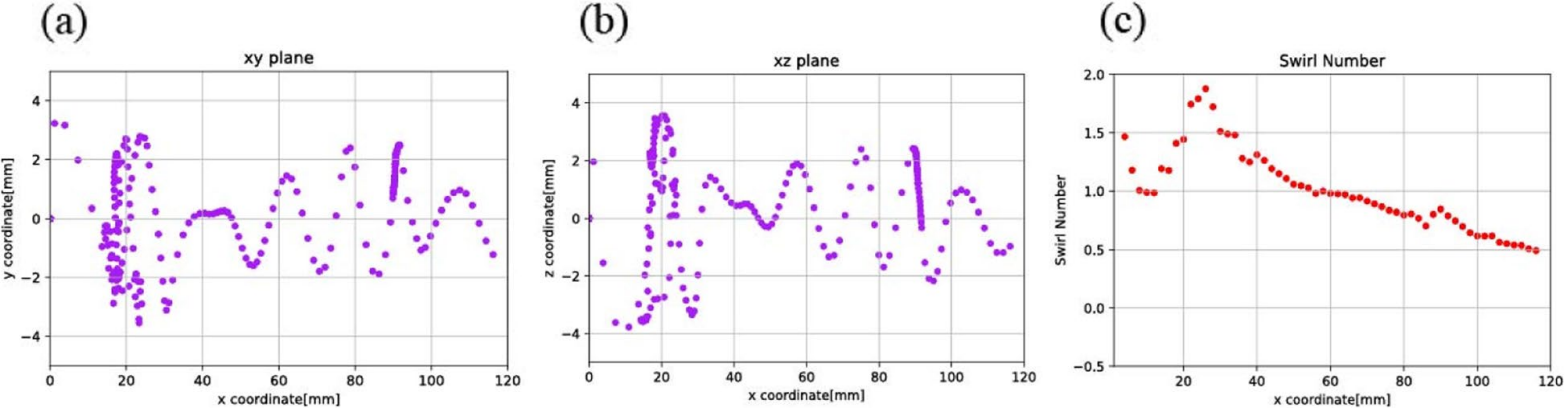
Moving path of the bubbles and Swirl numbers along the axial axis at inlet velocity: 2.97 m/s, viewed via (**a**) xy plane, **b** xz plane, and (**c**) Swirl number

**Fig. 6:**
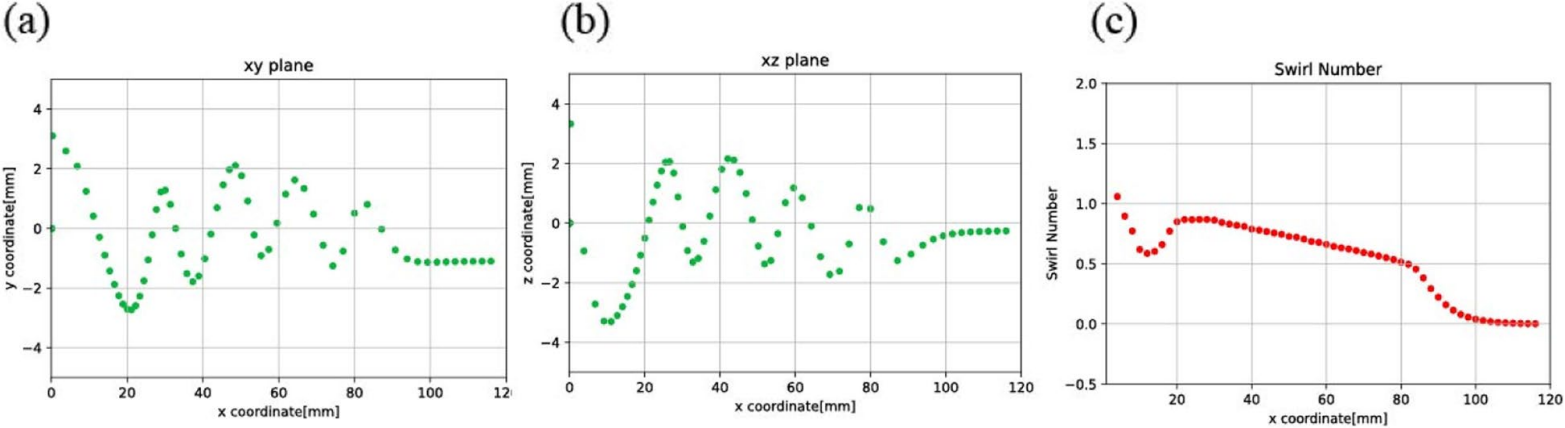
Moving path of the bubbles and Swirl numbers along the axial axis at inlet velocity: 0.0297 m/s, viewed via (**a**) xy plane, **b** xz plane, and (**c**) Swirl number

**Fig. 7:**
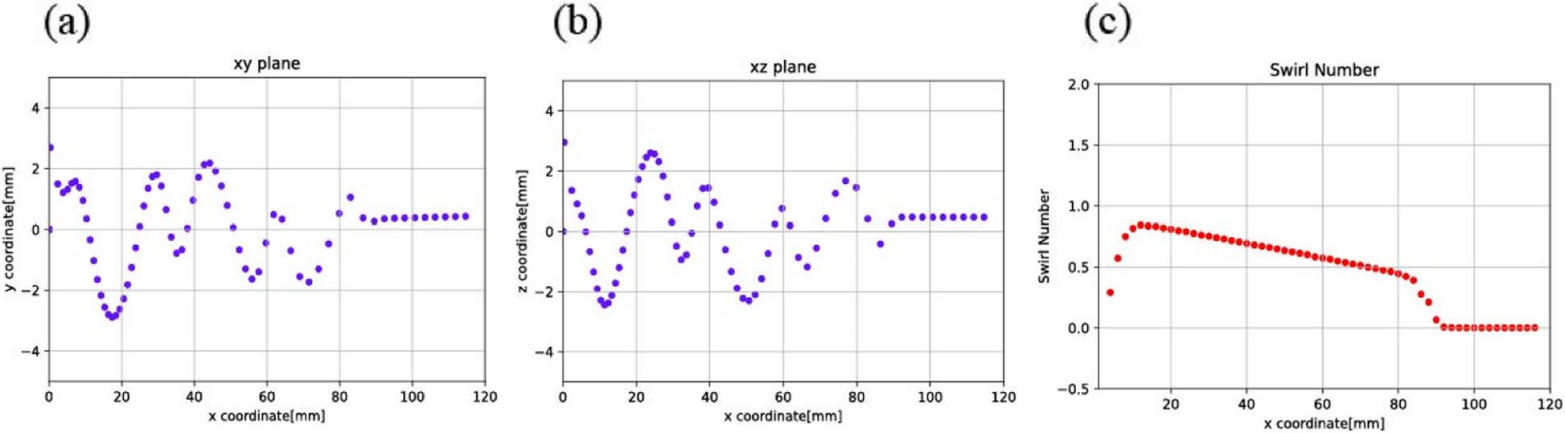
Moving path of the bubbles and Swirl numbers along the axial axis at inlet velocity: 0.00297 m/s, viewed via (**a**) xy plane, **b** xz plane, and (**c**) Swirl number

To pinpoint the effect of one specific parameter on the motion of the bubbles while traveling inside the bubble removing device is not easy. Thus, the Swirl number was chosen as one parameter. Simulation was analyzed based on the inlet velocity of Model 2, which was 0.297 m/s. The velocity of 0.297 m/s was determined based on the appropriate blood flow rate of 350 ml/min during hemodialysis and the typical size of the blood tube. Consequently, three other velocities: 2.97 mm/s (Fig. 5), 0.0297 mm/s (Fig. 6), and 0.00297 mm/s (Fig. 7) were selected, and then increased tenfold, decreased tenfold, and ultimately decreased 100 fold, respectively. It was found that the selected range of velocities produced a noticeable effect on the position of the bubbles. Forthwith, bubbles (size 10 µm) were released inside the model. Consequently, the path of the bubbles was observed.

In Fig. 5 (a,b), the randomly moving path of the bubbles was depicted. In Fig. 5 (c), on average, the Swirl number was higher than 1, and generated more bubbles. In Fig. 6 (a,b) and Fig. 7 (a,b), as velocity decreased, it was seen that the moving path of the bubbles converged in a straight line. In contrast, the non-thread region provided a forward flow instead of a swirling flow. The consequence of this fluid flow was that the bubbles moved forwards and passed the thread region. In Figs. 6(c) and 7(c), the Swirl numbers of both models were, on average, no greater than 1. In the non-thread region, the Swirl number reached zero, which meant there was no circular flow in that region.

## Discussion

**Fig. 8:**
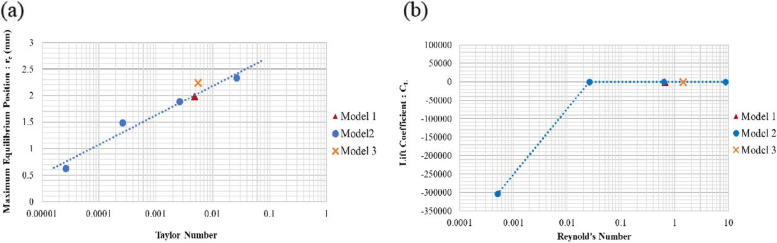
**a** Taylor number, and (**b**) Lift coefficient for Models 2 and 3 with different inlet velocities

Herein, the Swirl number along with the two major parameters: Taylor number and the Lift coefficient were discussed. It was noted that the maximum equilibrium position was directly proportional to the Taylor number. The Taylor number combined the phenomena of both Reynold’s number and Strouhal number. In Fig. 8 (b), the Lift coefficient having a low inlet velocity produced a negative Lift coefficient because of the low Reynold’s number. This phenomenon was known as the reverse Magnus effect. Further, a tiny wake i.e. the Strouhal number, was seen to occur behind the bubbles, causing an additional force in the opposite direction to the lift force. As a result, the collection of these forces was found to be greater than the lift force, and gave rise to a negative Lift coefficient [17]. The combined effect of Reynold’s number and Strouhal number resulted in a Taylor number. In Fig. 8 (a), it was seen that the Taylor number was determined at different inlet velocities. The changes in the Taylor number appeared to be in a linear relationship with the maximum equilibrium position. Hence, the Taylor number could be used to predict the position of the bubbles.

**Fig. 9:**
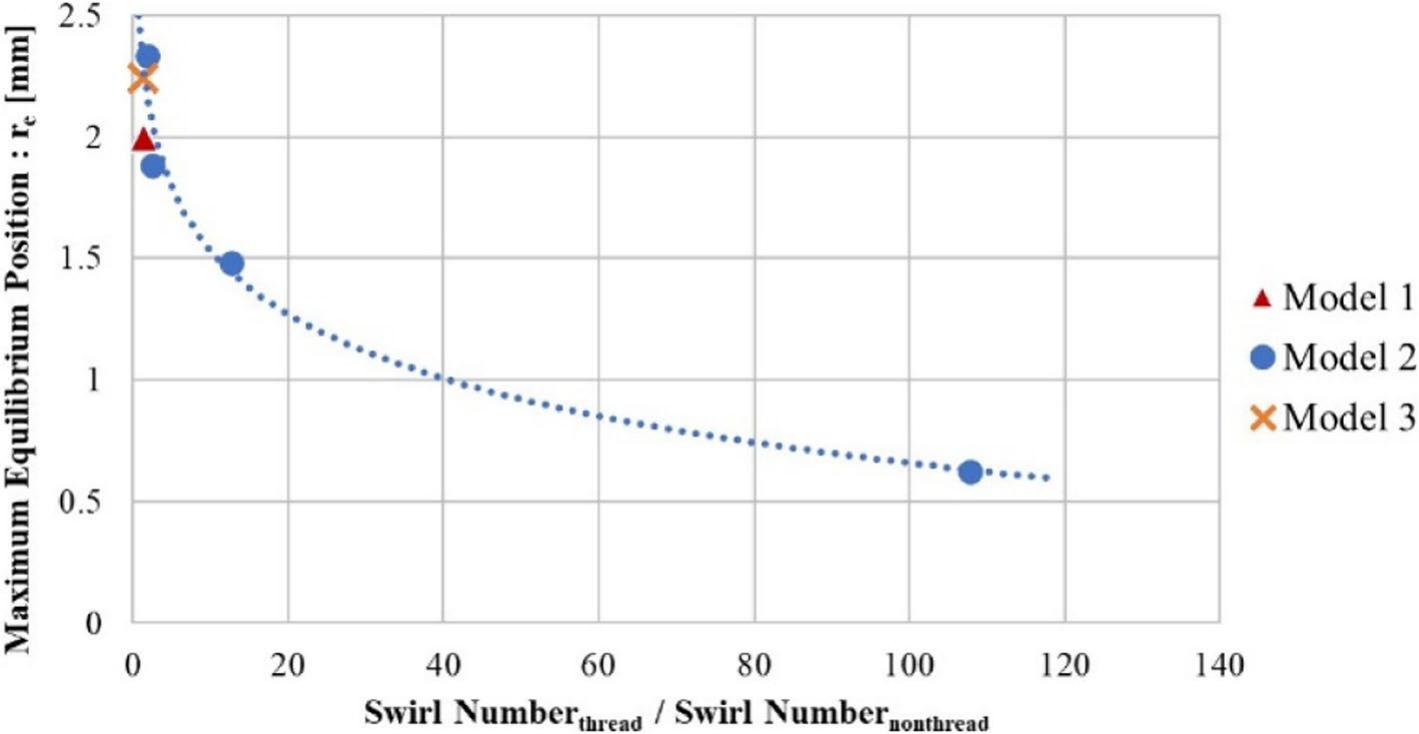
The relationship of the ratio of Swirl number to r_e_ (mm)

Despite the Taylor number and Lift coefficient, the Swirl number was also considered to support the phenomena of the thread design. In Fig. 9 when the inlet velocity decreased, it was seen that the swirling flow was reduced.

### Effect of size of pitch on the position of the bubbles

**Fig. 10:**
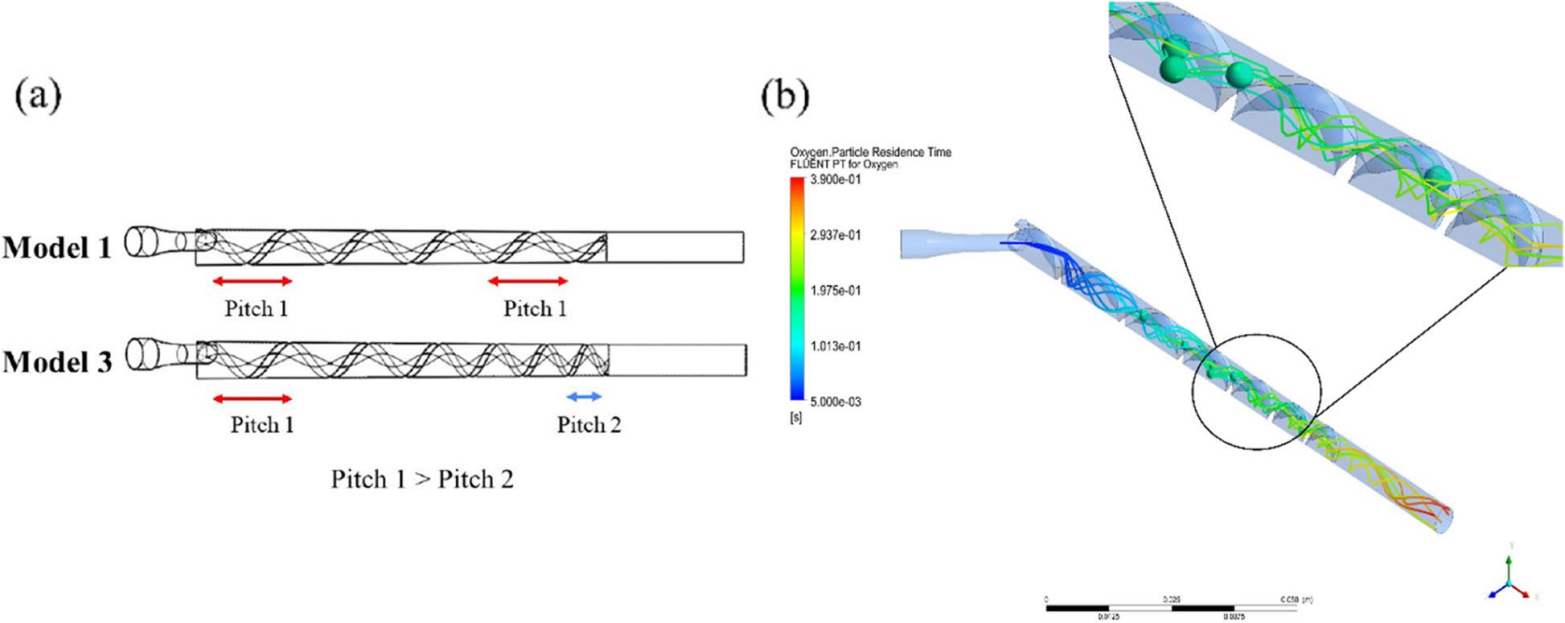
**a** Model 3 with the unequal pitched size, and (**b**) Simulation results of the unequal pitched model

In Fig. 10, when six bubbles (each 10 µm diameter) were released into the inlet pipe, both Model 3 and Model 1 provided similar results. Compared to Model 1, the Swirl number of Model 3 was found to be quite high, and more bubbles were generated. In Table 2, a comparison of the equilibrium position and the Swirl number of all three models was presented. Table 2 also showed the result of the unequally pitched Model 3 compared to Models 1 and 2.

**Table 2:** Comparison of the simulation results of Models 1, 2, and 3

	**Model 1**	**Model 2**	**Model 3**
r_e_ (mm)	1.995	1.883	2.243
Swirl number	0.736	1.028	0.998

**Fig. 11:**
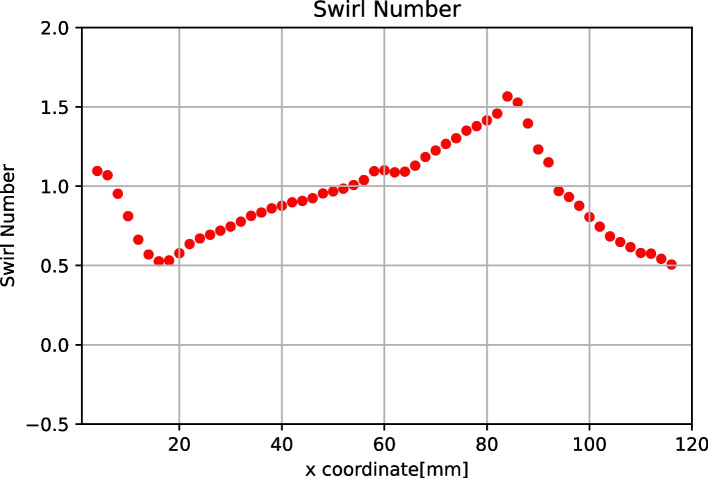
Swirl number along the axial axis of Model 3

As shown in Figs. 8 and 9, both the Taylor number and the Lift coefficient were similar for all three models. The maximum equilibrium position (r_e_) could be predicted by the previous trend as found in Models 1 and 2. The Taylor number could also be used to predict the motion of the bubbles. Model 3 demonstrated maximum equilibrium position of 2.243 mm, which proved to be the highest of all three models. In Fig. 11, however, it was found that the Swirl number was greater than 1, at the positions: 60 to 90 mm of the axial axis of the device. Therefore, Model 3 was seen to be unacceptable even if the unequal pitched thread could improve upon the collecting performance.

**Fig. 12:**
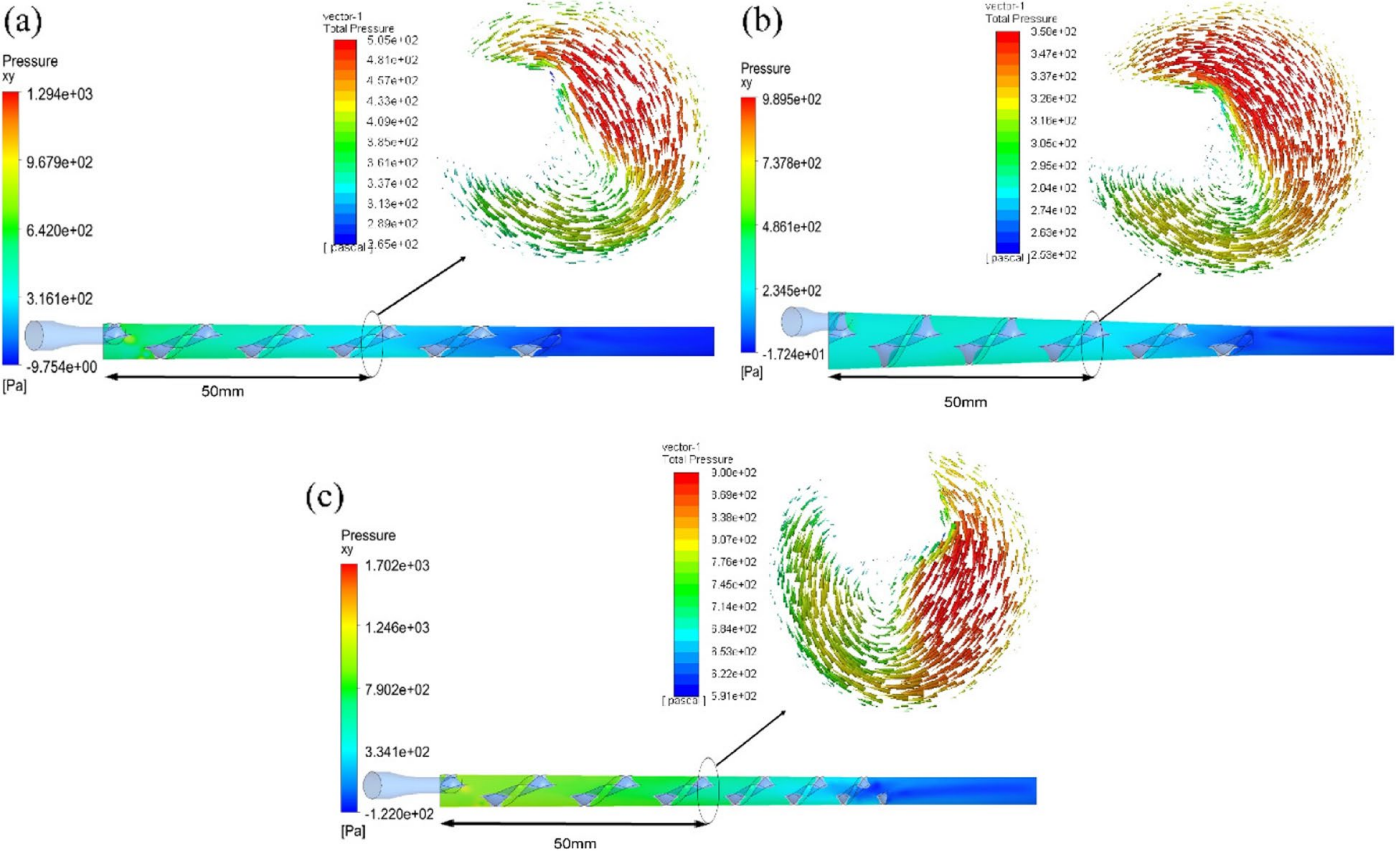
Pressure field inside the device of (**a**) Model 1, **b** Model 2, and (**c**) Model 3

In Fig. 12, the pressure fields of the fluid flow were presented for Models 1, 2, and 3. The cross-sectional pressure contours located 50 mm along the axial axis of the device were presented for all three models. It was acknowledged that low-pressure fields could lead to a core where bubbles agglomerate. Overall, Model 3 provided the highest pressure, followed by Models 1 and 2, respectively. While Model 2 had the lowest pressure inside the thread region of the device, it was found that the bubbles were more in the center area as compared to Model 1. However, it was seen that the pressure fields were not low enough to develop a core wherein the bubbles come together and gather in the center. It was significant that low pressure leads the bubbles to move to the core of the device. Nevertheless, the pressure after the thread was about the same. The fluid pressure, therefore, in the thread region mainly affects the position of the bubbles.

## Conclusion

In this paper, it is evident that Model 1 provided more maximum equilibrium positions of bubbles than Model 2. As such, the design of Model 1 caused the bubbles to move close to the wall of the device. Although Model 3 appeared to enhance the performance of the model, it generated more bubbles due to the high value of the Swirl number. Model 1, therefore, is seen to be a better choice for the design. For example, in Model 1, when 16,000 bubbles (typical situation of dialysis) were released into the inlet pipe, a total of 81.525% of bubbles were found in the radial region of 2.204 ± 0.297 mm. Different collecting planes were selected to analyze the position of the bubbles. It is clear that the number of bubbles were found mostly on the 100 mm plane of the axial axis. Hence, this plane was selected for collecting the bubbles around the wall region compared to the other two planes. The best model for elimination of bubbles proved to be Model 1.

## Methods

### Design model

**Fig. 13:**
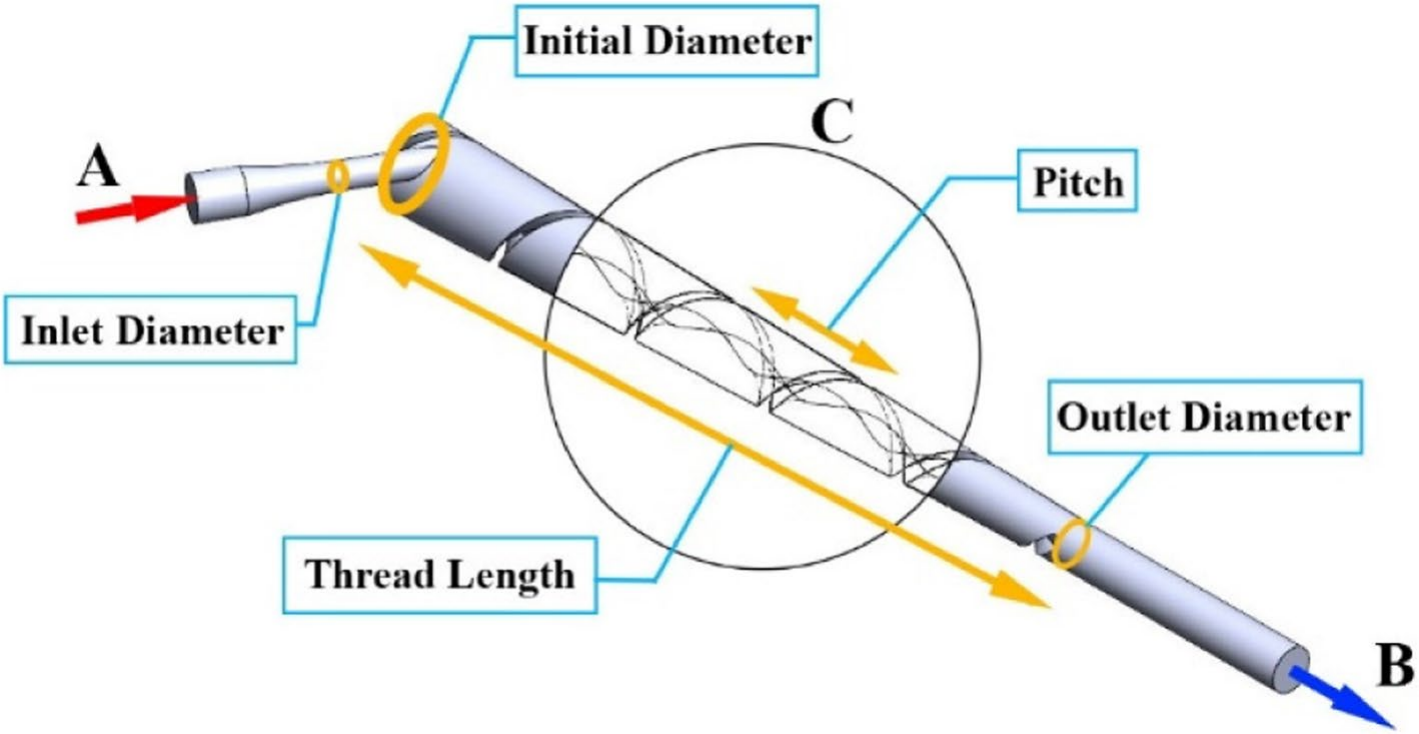
Overall design of the bubble removal device

The preliminary design (as shown in Fig. 13) is based on a cyclone design, which has a thread region C to support the circular flow. A is the inlet pipe that is connected to the dialyzer, and B is the outlet pipe that is connected to the returning tube. The diameter of the inlet pipe can be adjusted to obtain the desired diameter: 3 mm. In the thread region C, the initial diameter is different from the outlet diameter. The length of this thread region is the same for the three models, which is equal to 90 mm, and the pitch of the thread is defined as the distance between each thread. The flow velocity is determined, as in Eq. (7) to specify the inlet diameter and initial diameter and validate the complete swirling flow when the diameter of a typical blood tube is 5 mm [4]:

7 $${\text{V}}_{\text{initial}}\text{=}\frac{\text{r}_{\text{typ}}^2\cdot{\text{V}}_{\text{blood}}}{{({\text{r}}_{\text{initial}}\text{/2)}}^2}\text{,}$$

where V_initial_ (m/s) is the fluid velocity at the initial plane of the device, r_initial_ (m) is the radius of the initial inlet, r_typ_ (m) is the radius of a typical blood tube, and V_blood_ (m/s) is the fluid velocity in a typical blood tube.

In addition, the minimum pitch for supporting the swirling flow is calculated to specify the outlet diameter and pitch, as in Eq. (8):

8 $$\upphi \, \text{=}{\text{arcsin}}\sqrt{{(2({\text{P}}_{\text{outlet}}{\text{-P}}_{\text{initial}})/\rho \text{+}{{\text{V}}_{\text{outlet}}}^{2})/}{{\text{V}}_{\text{initial}}}^{2}}$$

where ϕ (degree) is the angle of the inlet pipe from the z-axis of the device, and P_initial_ (Pa) and P_outlet_ (Pa) are the pressure at the initial and pressure at the outlet of the device, respectively. The density of the fluid is ρ (kg/m^3^), and V_outlet_ (m/s) is the outlet velocity of the fluid.

The minimum requirement of the thread angle is illustrated, as in Eq. 8 and is equal to 21.13 degrees. This is the minimum angle that the design thread needs to support the flow. The design angle of the inlet pipe for the model is related to two parameters: the initial diameter of the device and the pitch of thread. As the helix shape of the thread design is in sine form, the mathematical operation can generate the relationship of these three parameters, as shown in Eq. (9):

9 $${\upphi }_{\text{design}} \, \text{=}{\text{arctan}}(\frac{2\uppi }{\text{Pitch}}\cdot \frac{{\text{D}}_{\text{initial}}}{2})$$

Where ${\upphi }_{\mathrm{design}}$ (degree) is the angle of the inlet pipe, which is calculated from the model, whereas Pitch (m) and D_initial_ (m) is defined as pitch length of the thread and the diameter of the front cross section of the device, respectively.

It is noted that the increase in the inlet pipe diameter and initial diameter provides a low average velocity. Decreasing the pitch and increasing the outlet diameter also provide the same result i.e. reduction in velocity. The Swirl number reveals an opposite trend compared to the outlet velocity; when velocity decreases, the Swirl number increases. However, the length of the thread region does not affect the velocity or the Swirl number if there are at least three thread cycles. In the design of the removal device, two models are seen to materialize: Model 1 and Model 2. These two models are different in their initial diameter. In Table 3, the design of Models 1 and 2 are outlined.

**Table 3:** Design variables of Model 1 and Model 2

Design variables	Model 1	Model 2
Inlet Diameter (mm)	3.00	3.00
Initial Diameter (mm)	6.00	10.00
Outlet Diameter (mm)	5.00	5.00
Pitch (mm)	18.00	18.00
Thread Angle (degree)	46.32	60.19
Length (mm)	90.00	90.00

Based on previous works wherein the unequal pitched model was found to increase the angular velocity [19, 20], the pitch region of Model 1 was developed. The improved model, Model 3, was designed with unequally pitched thread regions to enhance the model’s performance, as shown in Table 4.

**Table 4:** Pitch adjustment and length: Model 3

Length (mm)	Pitch (mm)
0 – 20	18.00
20 – 40	16.00
40 – 50	14.00
50 – 60	12.00
60 – 70	11.00
70 – 77	9.00
77 – 90	7.00

### Simulation

The simulation was conducted using Ansys 2020R1. The mesh independent method was applied prior to both steady-state and transient simulations, using a triangular type of mesh. The convergence test showed that the element size, which did not affect the velocity of fluid, was at least 0.55 mm and accounted for approximately 350,000 elements for steady state simulation. In the same way, transient simulation was also carried out alongside the convergence test, and the element size was found to be 0.38 mm, or 1,073,913 elements.

#### Material properties

Simulation consisted of 2 materials: blood and air. Since blood is a typically non-Newtonian fluid, blood behaves as a non-Newtonian fluid at a low shear rate position [13, 21], which rarely occurs in this simulation. For the most part, blood behaves like Newtonian fluid in the artery regions [21]. Thus, in this simulation, blood is assumed to be a Newtonian fluid. Next, air represents the bubbles. The properties of these materials are listed below, in Table 5.

**Table 5:** Material properties [13, 14, 22, 23]

Material	Density (kg/m^3^)	Dynamic Viscosity (kg/ms)	Surface Tension (N/m)
Air	1.225	1.72 × 10^–5^	5.4 × 10^–2^
Blood	1.05 × 10^3^	3.1 × 10^–3^	

#### Boundary Condition

In the simulation, the viscous model is characterized by a laminar flow due to the Reynolds number. The condition of the inlet velocity is 0.297 m/s, which is calculated via 350 ml/min of the proper fluid flow rate [9]; the outlet portion is followed by a pressure gradient. The model’s wall type is a stationary wall.

#### Discrete phase model (DPM)

DPM is applied to release the particles into the model. Since the relation of the Weber number and Ohnesorge number is in the less deformed region, the shape of the bubbles is spherical, which is the same as the particle shapes in the DPM method. The injected particle, whose property is air, replicates the bubble. The important setting for the DPM setting is the flow rate. The flow rate of the bubbles (each 10 µm in diameter) is set at 1.3 × 10^–13^ kg/s and 1.026 × 10^–9^ kg/s for bubbles (each 200 µm in diameter).

In this study, six bubbles are released into the model to present the overview of the bubbles’ movement. The releasing time for the bubbles was set at 0.025 s, while the time step size was 0.005 s. In addition, the study of 16,000 bubbles was observed to verify the overall model performance. For analysis of the 16,000 bubbles, simulation time was set at 1 s at the rate of 80 bubbles, for one-time step. The maximum iteration per one-time step was 300 and the residue became steady, approximately between 10^–3^ and 10^–5^.

## Data Availability

Data are available upon request. Please contact corresponding author at teeranoot.cha@kmutt.ac.th.

## Abbreviations

CFD: Computational fluid dynamics
DPM: Discrete phase model
